# Sonorensin: A new bacteriocin with potential of an anti-biofilm agent and a food biopreservative

**DOI:** 10.1038/srep13412

**Published:** 2015-08-21

**Authors:** Lipsy Chopra, Gurdeep Singh, Kautilya Kumar Jena, Debendra K. Sahoo

**Affiliations:** 1 Biochemical Engineering Research and Process Development Centre, CSIR-Institute of Microbial Technology, Sector-39A Chandigarh; 160036, India

## Abstract

The emergence of antibiotic resistant bacteria has led to exploration of alternative therapeutic agents such as ribosomally synthesized bacterial peptides known as bacteriocins. Biofilms, which are microbial communities that cause serious chronic infections, form environments that enhance antimicrobial resistance. Bacteria in biofilm can be upto thousand times more resistant to antibiotics than the same bacteria circulating in a planktonic state. In this study, sonorensin, predicted to belong to the heterocycloanthracin subfamily of bacteriocins, was found to be effectively killing active and non-multiplying cells of both Gram-positive and Gram-negative bacteria. Sonorensin showed marked inhibition activity against biofilm of *Staphylococcus aureus*. Fluorescence and electron microscopy suggested that growth inhibition occurred because of increased membrane permeability. Low density polyethylene film coated with sonorensin was found to effectively control the growth of food spoilage bacteria like *Listeria monocytogenes* and *S. aureus*. The biopreservative effect of sonorensin coated film showing growth inhibition of spoilage bacteria in chicken meat and tomato samples demonstrated the potential of sonorensin as an alternative to current antibiotics/ preservatives.

Bacteria in nature usually dwell in complex and dynamic surface-associated, sessile microbial communities called biofilms that are encaged in a self-produced extracellular polymeric substance (EPS), and create problems in clinical therapeutics^1^. Bacterial cells growing in a biofilm are physiologically diverse from planktonic cells of the same bacteria^2^, and the presence of EPS escalates antibiotic resistance by up to thousand folds^3^. Biofilms have immense negative impact on the world’s economy and pose severe problems to industry, public health and medicine^4^ due to increased rates of genetic exchange, altered biodegradability^5^, increased resistance to antibiotics and chemical biocides and increased production of secondary metabolites^4^,^5^,^6^. Many bacteria produce bioactive peptides or proteins called bacteriocins. Bacteriocins can be the next generation of antibiotics for combating multi-drug resistant and/or biofilm forming bacterial infections due to their different mechanisms of action, which include membrane-disrupting action, functional inhibition of proteins, binding with DNA, and detoxification of polysaccharides^7^. Some bacteriocins can be transferred in biofilm EPS through pores formed in the lipid component of the EPS, while others can disperse biofilms^7^.

In an infection, multiplying and non-multiplying bacteria exist side by side^8^. Non-multipliers are characterized by their lack of multiplication, survival in the presence of antibiotics and low metabolic activity^8^. It is also known that antibiotics kill multiplying bacteria and are inefficient at killing non-multipliers^9^, leading to slow or partial death of the target population in an infected tissue resulting in requirement of repeated doses of antibiotics. This extends the period of therapy and enhances the emergence of resistance. Targeting non-multiplying bacteria is a new approach to antibacterial therapy intended to swiftly destroy all of the non-multiplying and multiplying bacteria in an infection, thereby shortening antibiotic regimes that would slow the emergence of genetic resistance as mutation cannot occur if there are no live target bacteria^8^.

Numerous food preservation methods such as addition of preservatives (antibiotics, organic compounds such as sorbate, propionate, benzoate, acetate, and lactate), reduction of pH and water activity (acidification, dehydration) and thermal treatment (pasteurization, sterilization, heating) have been used to prevent food poisoning and spoilage^10^. Although these methods have been proven to be successful, however, consumers have been consistently concerned about the possible adverse health effects due to the presence of chemical additives in processed foods. This has led to the exploration of new means of preservation involving minimally processed foods with extended shelf-life^10^. For more than 50 years nisin produced by *Lactococcus lactis* has been used as a food preservative as it has been proven to be highly effective against microbial agents causing food poisoning and spoilage^11^.

Amalgamation of bacteriocins into packaging films to prevent food spoilage and to control pathogens has been an area of dynamic research for the last decade. Bioactive packaging film prevents microbial growth on food surface by direct contact of the package with the surface of foods, such as meats and cheese. It prolongs the lag phase and reduces the growth rate of microbes in order to extend shelf life and to maintain product quality^12^. There are two methods which have been commonly used to prepare packaging films with bacteriocins^13^. The first method incorporates bacteriocins directly into polymers (e.g., incorporation of nisin into biodegradable films^14^) and the other incorporates bacteriocins into packaging films by coating or adsorbing bacteriocins to polymer surfaces (e.g., nisin/methylcellulose coatings for polyethylene films, adsorption of nisin on polyamide, ethylene vinyl acetate, acrylics, polyester and polyvinyl chloride^13^). An *et al.* claimed that a polymer-based solution coating would be the most desirable method in terms of stability and adhesiveness of attaching a bacteriocin to a plastic film^15^.

Earlier, we have reported isolation, purification and characterization of sonorensin, a bacteriocin predicted to belong to heterocycloanthracin subfamily of bacteriocins from marine isolate *Bacillus sonorensis* MT93, and optimization of its production^16^,^17^. We have also reported the efficacy of sonorensin as a biopreservative in fruit products and as shelf life extender of pasteurised milk^17^. In the present study, the effectiveness of this bacteriocin as an anti biofilm agent and a food biopreservative has been demonstrated. We have also evaluated the effect of sonorensin against non-multiplying bacteria and an insight into its probable mode of action. It is the first bacteriocin of this subfamily to be characterized.

## Results

### Biofilm inhibition by sonorensin

**Figure 1:**
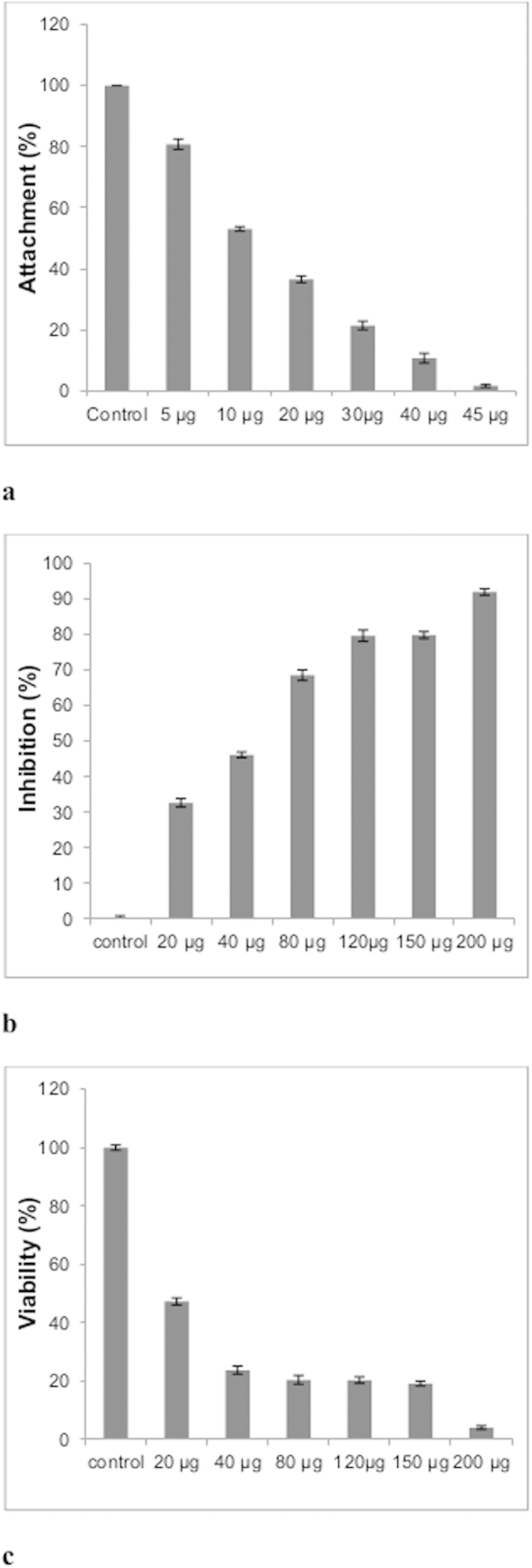
Effect of sonorensin on *S. aureus* biofilms. (**a**) Inhibition of attachment of *S. aureus* cells by sonorensin at various concentrations (**b**) Inhibition of biofilm formation (**c**) Viability of *S. aureus* cells biofilm (assayed by XTT). Control bars indicate *S. aureus* cells without any treatment taken as 100% in case of a & c and 0% in case of b. Each well of 96 -well plate contains 4 × 10^6^ CFU *S. aureu*s cells and variable concentration of sonorensin in 200 μl of BHI-sucrose. The plates were incubated at 37 °C for 4 h and 24 h for biofilm attachment assay (**a**) and inhibition of biofilm formation (**b**), respectively. (**a**) About 1.8 ± 0.05% attachment of biofilm was observed in the presence of 1X MIC of sonorensin. (**b**) Sonorensin showed significant inhibitory activity against *S. aureus* biofilm formation at 24 h, (**c**) Reduced XTT conversion was observed in wells with higher sonorensin concentrations and the control (without treatment) showed maximum reduction of XTT. The results were presented as mean ± SD and differences between the control and treated samples were statistically significant (n = 3) (p < 0.005).

When different concentrations of sonorensin were incubated with *S. aureus* for 4 h at 37 °C for adherence to the wells of microtiter plates, it inhibited biofilm attachment in a concentration dependent manner (Fig. 1a). About 1.8 ± 0.05% attachment of biofilm was observed in the presence of 1X MIC (~50 μg/ml) of sonorensin. Sonorensin showed significant inhibitory activity against *S. aureus* biofilm formation at 24 h, in relation to its concentration (Fig. 1b). When the sonorensin treated biofilms were subjected to 2, 3-Bis (2-methoxy-4-nitro-5-sulfophenyl)—2H-tetrazolium-5-carbox-anilide (XTT) assay, reduced XTT conversion was observed in wells with higher concentration of sonorensin while the negative control showed the maximum reduction of XTT indicating the effect of sonorensin on the viability of cells in biofilm (Fig. 1c).

**Figure 2:**
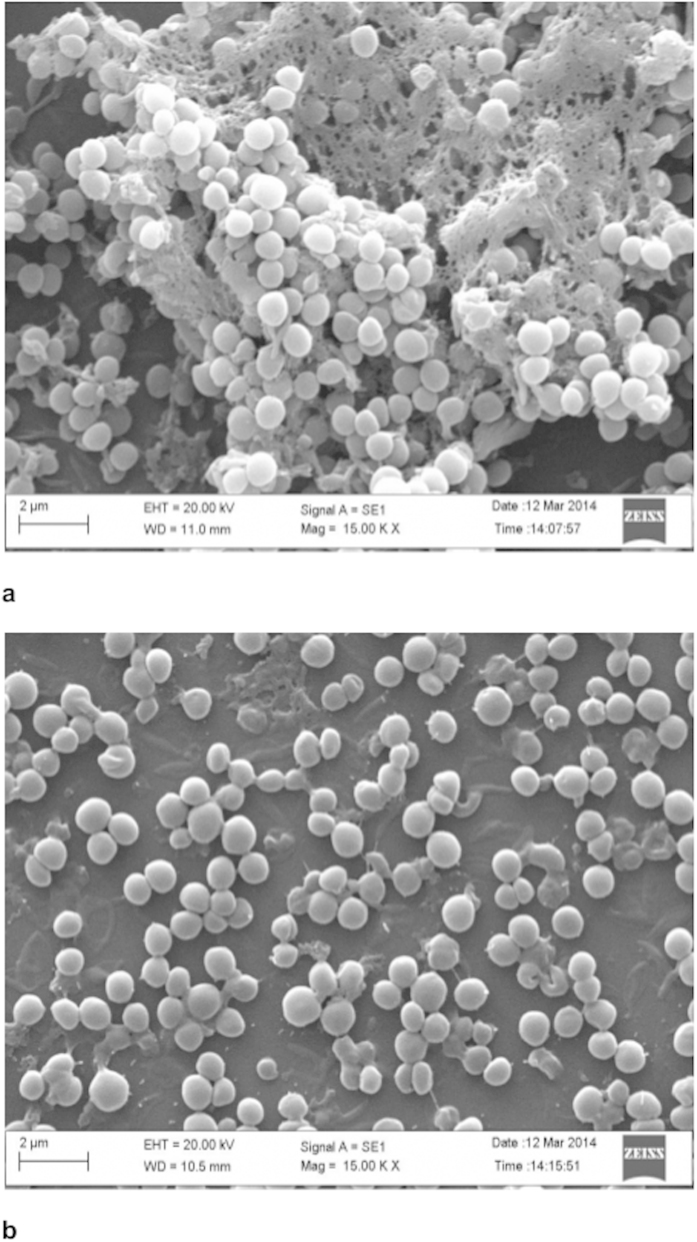
The scanning electron micrographs of mature (48 h old) biofilm of *S. aureus* cells (**a**) without sonorensin treatment and (**b**) after sonorensin treatment (50 μg/ml). The biofilms without sonorensin treatment consisted of nearly uniform, thick layer of cells embedded within a self produced matrix whereas thinning of mature biofilms was observed after sonorensin treatment.

Scanning electron micrographs of biofilms of *S. aureus* formed on cover slips and the effect of sonorensin on the preformed biofilm are shown in Fig. 2. For the non-treated controls, a biofilm formed consisted of nearly uniform, thick layer of cells (Fig. 2a), while the biofilm treated with sonorensin (50 μg/ml) was much less dense, and individually formed colonies could be seen (Fig. 2b).

### Sonorensin is effective against non-multiplying bacteria

**Figure 3:**
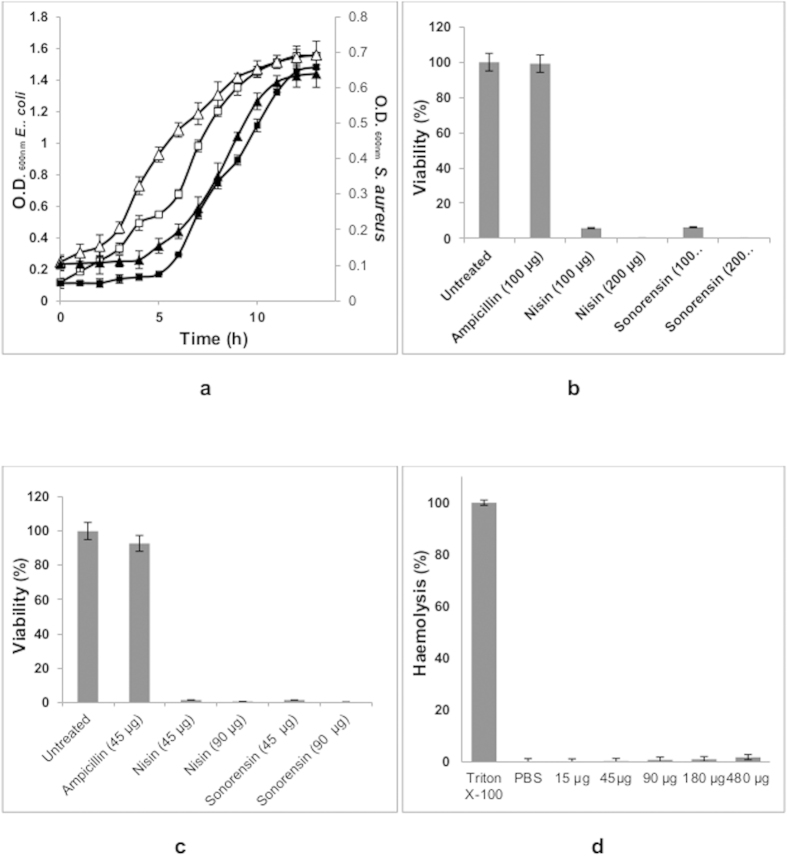
Effect of sonorensin on non-multiplying bacteria. (**a**) Comparative growth curves of active (open symbols) and non-multiplying (filled symbols) cells of *E. coli* (squares) and *S. aureus* (triangles). Cell viability was used as a measure of the effect of sonorensin on non-multiplying cells of (**b**) *E. coli* and (**c**) *S. aureus*. The CFU count from the untreated sample was taken as 100% in the cell viability calculations. An extended lag phase of non- multiplying cells compared to their vegetative counterparts (**a**). The sensitivity to nisin and tolerance to ampicillin confirmed non-multiplying state of cells. Sonorensin was effective against non-multiplying cells of both *E. coli* (**b**) and *S. aureus* (**c,d**) Sonorensin toxicity to mammalian cells was assayed by measuring its haemolytic activity. Triton X-100 and PBS served as controls. Sonorensin, at concentrations toxic to vegetative and dormant cells of *E. coli* and *S. aureus*, had virtually no effect on RBCs and only 1.7 ± 0.04% haemolysis was observed at higher concentrations of sonorensin. All the experiments were repeated three times in triplicate. The results were presented as mean ± SD and differences between the control and treated samples were statistically significant (n = 3) (p < 0.005).

Sonorensin was investigated for its efficacy against bacterial cells in dormant stage. *Escherichia coli* and *S. aureus* were used as indicator strains to produce long duration stationary phase cells of Gram-negative and Gram-positive bacteria, respectively. An extented lag phase compared to their vegetative counterparts in a regrowth experiment (Fig. 3a), sensitivity to nisin (Fig. 3b,c) and tolerance to ampicillin (Fig. 3b,c) confirmed their non-multiplying state. Susceptibility of vegetative cells of both *E. coli* and *S. aureus* to ampicillin is shown in Supplementary Fig. S1 online. The revived cultures regained sensitivity to antibiotics signifying that no further antibiotic resistance was attained through any genetic alteration during dormancy. Sonorensin was found to kill non-multiplying cells of both *E. coli* and *S. aureus* (Fig. 3b,c). On comparing its antimicrobial activity with nisin, it was observed that sonorensin was as effective as nisin in killing non-multiplying cells of *S. aureus* and *E. coli*.

As the MIC of sonorensin against both the vegetative and non-multiplying cells of *E. coli* was higher as compared to that of *S. aureus*, the inhibitory activity of treatment combining sonorensin and the chelating agent EDTA on *E. coli* was investigated. It was found that sonorensin in combination with 20 mM EDTA showed more antimicrobial activity against both multiplying and non-multiplying cells of *E. coli* as compared to sonorensin alone (see Supplementary Fig. S2 online). The controls, EDTA and buffer individually, did not show any activity.

The haemolytic activity (if any) of sonorensin was determined by testing its toxicity against mammalian cells. It was found that sonorensin, at concentrations at which it destroys vegetative and dormant cells of *E. coli* and *S. aureus*, had virtually no effect on red blood cells (RBCs) and only 1.7 ± 0.04% haemolysis was observed at high concentrations of sonorensin (Fig. 3d).

### Mode of bactericidal action: increased cytoplasmic membrane permeability

**Figure 4:**
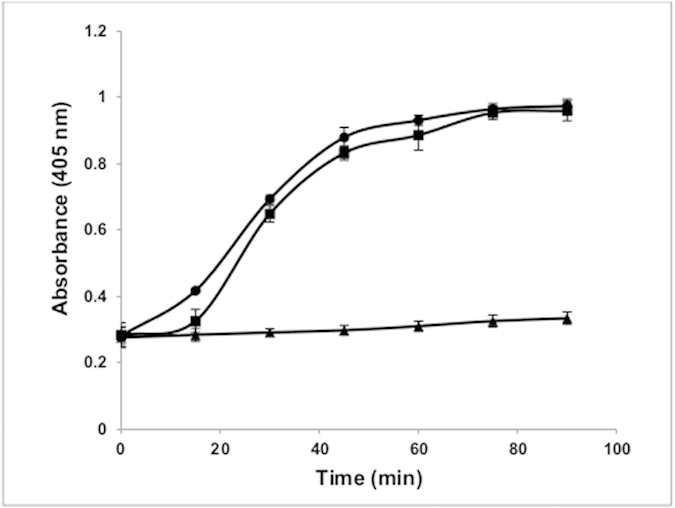
The cytoplasmic membrane permeabilization of *S. aureus* cells treated with sonorensin (squares) and nisin (circles). The untreated *S. aureus* cells (triangles) were taken as control. When the cytoplasmic membrane was permeable ONPG entered the cytoplasm and degraded by β-galactosidase, producing O-nitrophenol that showed absorbance at 405 nm. Sonorensin induced an increase in the permeability of *S. aureus*. The experiment was carried out three times in triplicate. The results were presented as mean ± SD and differences between the control and treated samples were statistically significant (n = 3) (p < 0.005).

When the cytoplasmic membrane is permeable, ortho-Nitrophenyl-β-galactoside (ONPG), a non membrane—permeative chromogenic substrate, enters the cytoplasm and is degraded by β-galactosidase, producing O-nitrophenol that shows absorbance at 405 nm^18^. As shown in Fig. 4, sonorensin induced an increase in the permeability of *S. aureus* cytoplasmic membrane over time and in case of nisin (at same concentration), which has almost same MIC value against *S. aureus*, produced similar results of permeability. This suggested that sonorensin could permeabilize the cytoplasmic membrane of *S. aureus*.

**Figure 5:**
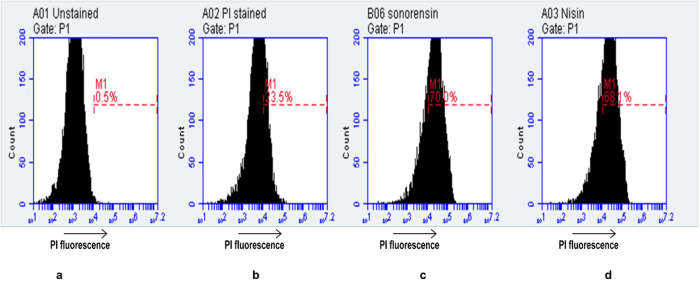
Flow cytometry analysis of effects of sonorensin and nisin on membrane integrity of *S. aureus* cells. Data were displayed as flow cytometric histograms of counted bacterial events (y-axis) associated cell fluorescence (x-axis). Marker M1 is the region that the damaged cells were stained by PI. (**a**) Unstained *S. aureus* cells (**b**) Untreated, PI stained *S. aureus* cells (**c**) Sonorensin treated, PI stained *S. aureus* cells, (**d**) nisin treated, PI stained *S. aureus* cells. For each sample 10^4^ cells were analysed. The membrane integrity of *S. aureus* cells was destroyed by treatment with sonorensin.

PI is a viability-fluorescent marker that can penetrate impaired cells and intercalate into nucleic acid^18^. Sonorensin and nisin induced membrane damage of *S. aureus* cells was determined by staining the cells with PI after treatment with the sonorensin and nisin at 37 °C for 1 h using Flow Cytometer. As shown in Fig. 5, in the absence of any antibacterial agent, 76.5% of untreated control *S. aureus* cells showed no PI fluorescence signal. However, a significant increase in PI fluorescence was observed for the cells treated with sonorensin (70.0% cells stained with PI) and nisin (68.1% cells stained with PI) as depicted in Fig. 5. These results indicated that the membrane integrity of *S. aureus* cells was destroyed by treatment with sonorensin and the effectiveness was comparable to that of nisin.

**Figure 6:**
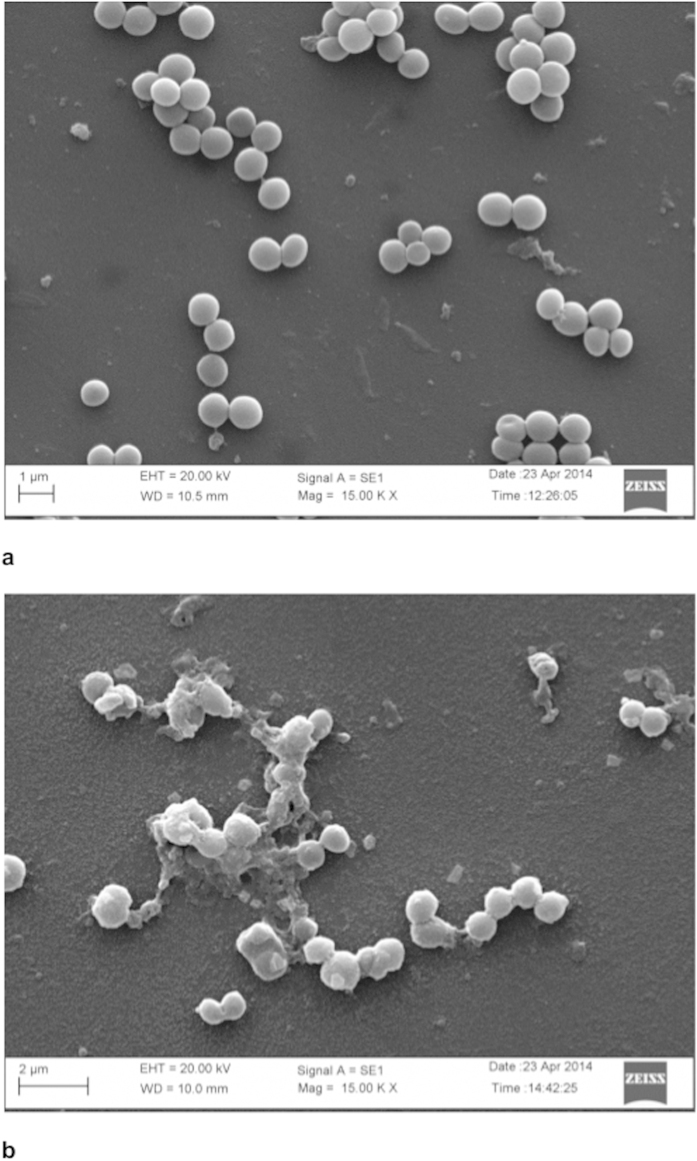
The scanning electron micrographs of *S. aureus* cells (**a**) without sonorensin treatment, and (**b**) after sonorensin treatment (50 μg/ml) for 4 h. The treatment of *S. aureus* with sonorensin displayed roughening of cell surface with cell debris while smooth cell surface was observed in cells without treatment.

To further gain insight into the mode of bactericidal action of the sonorensin, SEM of *S. aureus* treated with lethal dose of sonorensin was performed. When compared to untreated cells, *S. aureus* cells pre-incubated with 50 μg/ml of purified sonorensin for 4 h displayed major alterations like roughening of the cell surface with accumulation of cell debris and cell lysis (Fig. 6).

### Bioactive polyethylene film

**Figure 7:**
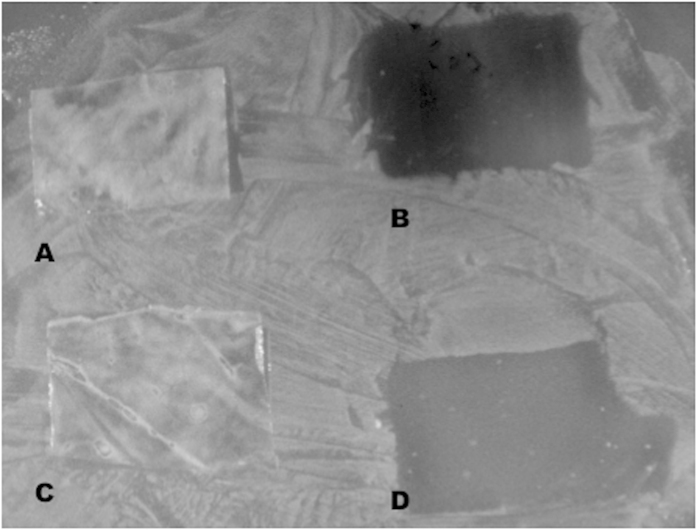
Inhibitory activity of coated LDPE films against *S. aureus*. (**a,c**) control; (**b**) with sonorensin coated LDPE film; (**d**) with nisin coated LDPE film. The growth of *S. aureus* was inhibited by sonorensin and nisin coated LDPE films whereas *S. aureus* grew homogeneously on the surface of the plate and underneath the untreated LDPE film used as control.

The low density polyethylene film (LDPE), coated with sonorensin and nisin, showed inhibitory activity against *S. aureus* (Fig. 7). Untreated film did not show any antimicrobial activity. As shown in Fig. 7, the superficial growth of indicator strain was limited to the area surrounding the activated film that could clearly inhibit the development of the *S. aureus* in contrast, it could grew homogeneously on the surface of the plate and underneath the untreated film used as control.

**Figure 8:**
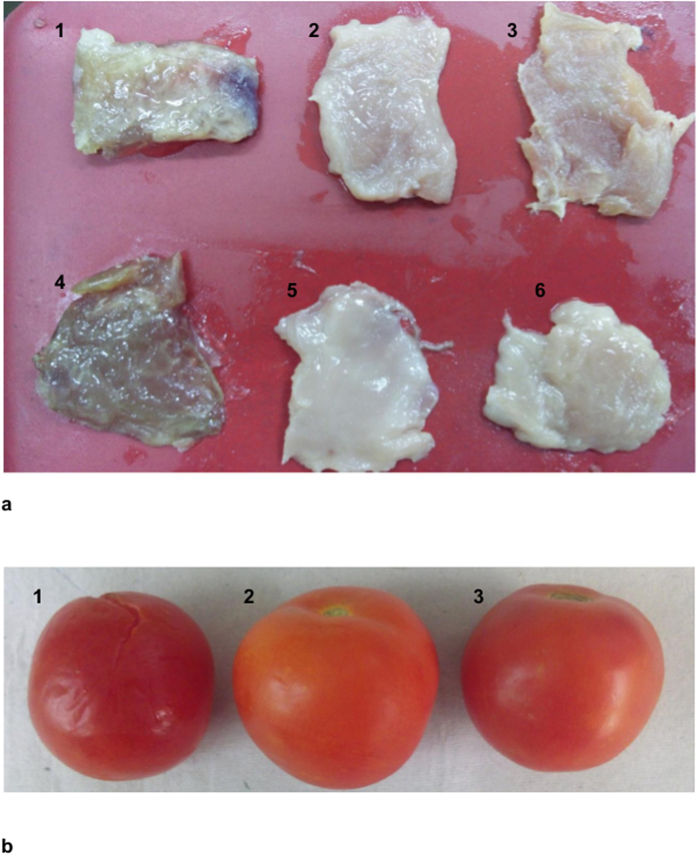
Preservative effect of coated LDPE film during the storage of (**a**) meat (**b**) tomatoes. (**a**) Meat samples were spiked with *L. monocytogenes* (1–3) and *S. aureus* (4–6). Spoilage of meat is visible in meat samples packaged in control LDPE films (1 & 4) whereas no spoilage was observed in samples packaged with sonorensin (2 & 5) and nisin (3 & 6) coated LDPE films. (**b**) Tomato sample (1) packaged in untreated LDPE films showed signs of spoilage in contrast to no spoilage in case of tomatoes packaged in sonorensin (2) and nisin (3) coated LDPE films.

The sonorensin and nisin coated LDPE films were checked for their efficacy to inhibit the growth of food spoiling bacteria such as *S. aureus* and *L. monocytogenes*. Fresh meat spiked with these organisms and tomatoes were packed in sonoresin and nisin coated LDPE films and untreated LDPE films (control). The spoilage of both meat and tomatoes was observed in case of untreated packaging films after 4 days and 7 days of incubation at 4 ^o^C respectively (Fig. 8). However, no signs of spoilage were seen in meat and tomatoes packed with sonorensin and nisin coated films (Fig. 8) even after 15 days of storage at refrigerated conditions. Moreover foul/stinky smell that was observed in meat samples packed with untreated films was absent in meat packed with sonorensin and nisin coated films. This suggested that like nisin, sonorensin could also be used as a food bio-preservative.

## Discussion

The growth of biofilms is a significant problem within the healthcare and food industries. The characteristic resistance offered by biofilm-associated communities of microorganisms leading to their persistent survival is an important challenge to address. Biofilms have been attributed to food-borne illnesses^19^,^20^ and can also cause premature biofouling in dairy and other processed foods. Though, many antibiotics are effective against planktonic cells, fewer are active against biofilms^21^. Furthermore, while it is suggested that bacteriocins may inhibit the development of biofilms^22^, their effect upon microbial cells in a biofilm is not fully understood. When applied on preformed *S. aureus* biofilms, sonorensin was able to significantly reduce biofilm cell viability even at lower concentrations as indicated by XTT assay (Fig. 1c). SEM analysis of the biofilms also indicated that sonorensin not only inhibited biofilm formation but also caused thinning of mature biofilms (Fig. 2a,b).

The fall in the emergence of new antimicrobials in the market during the past two decades is worrying, particularly in view of the rise in bacterial resistance against many of the currently used antibiotics. Shifting the aim of drug development from multiplying to non-multiplying bacteria is expected to generate a new set of prospectives for antibiotic development and could result in the development of the drugs that would shorten the duration of therapy^8^,^23^. In addition to possessing a broad spectrum of antimicrobial activity, sonorensin also showed promising antimicrobial action against non-multiplying bacteria, which constitute a major cause of recurrence of chronic infections. The activity of sonorensin against non-multiplying cells of *S. aureus* was comparable to nisin. However, in case of non-multiplying cells of *E. coli*, sonorensin was effective at a comparatively higher concentration. The effectiveness of sonorensin against non-multiplying cells of *E. coli* was increased in combination with 20 mM EDTA that indicated the effects of sonorensin in reducing population being facilitated by the chelation of Mg^2+^ ions, present in the outer membrane of *E. coli*, by EDTA. This is in accordance with the reports that the removal of Mg^2+^ ions from the lipopolysaccharide layer of the outer membrane results in the loss of lipopolysaccharide and an increase in cell permeability^24^. This increase in outer membrane permeability is proposed to facilitate inactivation of the cell by sonorensin action at the cytoplasmic membrane. Similar results of the effect of EDTA on the activity of nisin against gram negative bacteria *Salmonella* spp. have been reported by Stevens *et al.*^25^. The activity of sonorensin against non-multiplying cells suggested that the bacterial resistance would not develop against sonorensin as all the bacterial cells (multiplying as well as non-multiplying) would be killed and no bacteria would survive that could evolve and develop resistance. Hu *et al.* reported an antibiotic, called HT61, active against non-multiplying bacteria, including methicillin resistant and sensitive *S. aureus*^23^.

The membrane is the main barrier that limits the distribution and entry of antibiotics^18^. In addition to antimicrobial activities, bacteriocins serve as an anti-resistance compound to classic antibiotics as they are able to interact with bacterial membranes, create ion permeable channels leading to increased cytoplasmic membrane permeability and hence, bacterial cell death^26^. In case of sonorensin, it showed permeabilization effect on *S. aureus* membrane and increased the plasma membrane permeability for influx of ONPG into cells. Moreover the investigation of sonorensin treated cells stained with PI also revealed the influx of PI into the cells indicating that the cytoplasmic membrane could be the most probable target of action of sonorensin. In this sense, sonorensin could be the potential candidate for therapeutics as it does not target the cell components like nucleic acids and proteins which are the major targets of most antibiotics, leading to the targeted bacteria developing resistance against such antibiotics.

Antimicrobial packaging is a promising form of active food packaging and an emerging technology. Antimicrobial packaging film prevents microbial growth on food surface by direct contact of the package with the surface of foods. For this reason, the antimicrobial packaging film must be in contact with the surface of the food so that bacteriocins can diffuse to the surface^13^. When the food product is packaged with such films the antimicrobial substance is released slowly onto the surface of the food product thus providing protection for extended periods^27^.

The results of present study of antimicrobial packaging applications on meat are quite promising. The bioactive packaging film of sonorensin showed its biopreservative effect during the storage of meat for long duration. Similar results of coating of LDPE films with bacteriocins have been previously reported^15^,^28^,^29^. Dawson *et al.* evaluated the effect of lauric acid and nisin impregnated soy based films on the growth of *L. monocytogenes* on turkey Bologna^30^. Ming *et al.* developed pediocin-coated casings that showed useful in controlling the growth of *L. monocytogenes* in meat and poultry products^31^. Sonorensin active packaged film also showed preservative effect upon storage of vegetables. These results indicated sonorensin to be a promising biopreservative agent and its incorporation in films may control the growth of undesirable bacteria, thereby extending the shelf life and enhancing the microbial safety of food products.

## Materials and Methods

### Reagents and media

Nisin and Histopaque-1077 were procured from Sigma- Aldrich Inc. (St. Louis, MO, USA) and microtitre plates were procured from Nunc (Nalge Nunc International, Denmark). BD vacutainer was obtained from BD biosciences (BD biosciences, CA, USA). All other reagents and media components used were either of analytical grade or of highest purity grade available in India.

#### Bacterial strains

*Bacillus sonorensis* MT93 (accession number HF944961.1) was isolated from marine soil sample collected from Parangipettai, India as reported previously^16^. Indicator strains: *B. subtilis* (MTCC 121), *S. aureus* (MTCC 1430), *E. coli* (MTCC 1610) and *L. monocytogenes* (MTCC 839) were procured from Microbial Type Culture Collection (MTCC), Chandigarh, India.

### Minimal inhibitory concentration (MIC)

The MIC of sonorensin against *E. coli*, *S. aureus* and *L. monocytogenes* was determined as reported previously^16^.

### Biofilm formation

The overnight culture of *S. aureus* was diluted to ~2 × 10^7^ CFU/ml. 200 μl of this suspension and 1% sucrose was added to wells of 96-well plates and incubated at 37 °C for 24 h. The negative control was un-inoculated media processed similarly. After incubation, the spent media was aspirated gently and wells were washed with 250 μl of PBS to remove planktonic bacteria and air-dried. 200 μl of 99% (v/v) methanol was added and incubated for 15 min for fixation and aspirated, and plates were allowed to dry. Wells were stained with 200 μl of 0.1% (v/v) crystal violet for 5 min. Excess stain was gently rinsed off and plates were air-dried. Stain was resolubilized in 200 μl of 95% (v/v) ethanol and cell concentration was measured at OD_595_ nm^32^.

### Biofilm attachment assay and inhibition of biofilm formation

Biofilm attachment assay and inhibition of biofilm formation were performed as described previously^33^,^34^. The overnight culture of *S. aureus* (diluted to ~2 × 10^7^ CFU/ml) was added to wells of 96-well plates with different concentrations of sonorensin (in triplicate). The plates were incubated at 37 °C for 4 h and 24 h for biofilm attachment assay and inhibition of biofilm formation, respectively. The positive control was *S. aureus* in Brain heart infusion (BHI) -sucrose medium without sonorensin. After the incubation, wells were washed with PBS and the cell concentration was measured at OD_595_ nm^34^.

After sonorensin treatment and incubation period of biofilm formation, XTT reduction assay was performed as a measure of metabolic activity in order to estimate viable cells. The wells were washed with PBS, and 20 μl of XTT (500 mg/ml) was added and incubated for 2 h at 37 °C. The color developed was read at 495 nm in a plate reader.

### SEM

To examine the anti-biofilm activity of sonorensin by microscopy, *S. aureus* biofilm was developed on poly (L-lysine) coated cover slips. Following addition of sonorensin (50 μg/ml), cover slips were incubated for 24 h. The samples were then fixed with Karnovsky’s fixative^35^ for 2 h at 4 ^o^C, washed with phosphate buffer, dehydrated with a graded ethanol series and finally by tert- butyl alcohol. Then, the cover slips were dipped in tert- butyl alcohol, kept at −20 ^o^C followed by freeze drying and platinum coating. The samples were observed using Zeiss EVO 40 instrument (Ukraine).

### Effectiveness of sonorensin against non-multiplying bacteria

#### Non-multiplying cell preparation

*S. aureus* and *E. coli* cells were grown in 50 ml LB medium at 37 °C and 200 rpm for 7 days^23^. The 7-day-old cultures were centrifuged and washed twice with physiological buffered saline (PBS, pH 7.2), and following resuspension in same buffer were incubated at 37 °C and 200 rpm for 7 days. Cell viability was checked by counting colony-forming units (CFUs) every 24 h. The culture showed a decrease in CFU/ml in the first 4 days, and remained constant thereafter, at 3.18 × 10^5^ and 4.2 × 10^6^ CFU/ml for *S. aureus* and *E. coli*, respectively. The cells after incubation for 4 days were analysed for dormancy state by overnight treatment with lethal dose of ampicillin (2 mg/ml) and by comparison of regrowth curves with that of vegetative cells and were used as stocks of non-multiplying cells. Regrowth was performed in 200 μl LB medium in 96 well plate and OD_600_ nm was monitored.

#### Antimicrobial activity assay against Non-multiplying cells

Different antimicrobial agents (ampicillin, nisin and sonorensin) at concentration range of 50 μg/ml–200 μg/ml were added to 100 μl of non-multiplying cell suspension of *S. aureus* and *E. coli* in 96 well plate. Cells were incubated at 37 °C and 200 rpm overnight, and then washed with PBS to remove excess antimicrobial agents and then plated in triplicate on BHI agar plates for estimation of CFU. An untreated sample, taken as control, was processed in the same way.

### Effect of EDTA on sonorensin activity

Effect of EDTA on sonorensin activity against *E. coli* was tested in the presence of 20 mM EDTA, as described previously^25^ (see Supplementary methods online). Sonorensin, 20 mM EDTA, buffer and sonorensin in combination of 20 mM EDTA were assayed for antimicrobial activity.

### Haemolytic activity assay

The haemolytic activity was measured on human red blood cells (RBCs) as reported previously^36^ and the protocol was approved by the Ethics Committee of the Institute. Complete lysis was measured by suspending RBC’s in 1% Triton X-100^37^ (see Supplementary methods online).

### Membrane damage of bacterial cells

#### Cytoplasmic membrane permeability

The cytoplasmic membrane permeabilization by sonorensin and nisin was investigated by using ONPG and measuring β-galactosidase activity in cells as described previously^38^ (see Supplementary methods online). The hydrolysis of ONPG to O-nitrophenol over time was monitored at 405 nm with a microplate reader (Biotek, USA).

#### Flow cytometry

The experiment was performed according to Joshi *et al.* with some modifications^37^. *S. aureus* cells were collected in mid exponential phase, washed three times with phosphate buffered saline, and resuspended at a concentration of 1 × 10^6^ CFU/ml in the same buffer. This was followed by addition of 50 μg/ml of sonorensin and nisin and incubation at 37 °C for 1 h. Then, the mixtures were incubated with PI solution (10 μg/ml) for 30 min at 4 °C in the dark. PBS was used as negative control. Flow cytometry analysis was conducted using Accuri C6 Flow Cytometer in FL2 channel. For each sample 10^4^ cells were analysed. Data was analyzed by C Flow Plus software (Becton Dickinson, San Jose, CA, USA).

#### SEM

To examine the bactericidal activity, *S. aureus* cells were grown in NB to an exponential phase and harvested by centrifugation. The pellet obtained was resuspended in fresh NB and aliquots of 5 ml containing about 1 × 10^6^ cells/ml were incubated at 37 °C for 4 h with sonorensin (50 μg/ml). Samples withdrawn at 4 h were centrifuged and pellets were resuspended in 500 μl of phosphate buffer. Each sample was spread on a poly (L-lysine)-coated glass slides, fixed and observed with a Zeiss EVO 40 instrument.

### Antimicrobial polyethylene films preparation

Sonorensin and nisin were coated on LDPE as described previously^39^. The antimicrobial coating mixtures were prepared by adding 2 g of methyl cellulose to 10 ml of sonorensin and nisin (500 μg/ml) separately. The mixtures were then homogenised at 16,000 rpm for 2 min in a homogenizer (Silent crusher M, Heidolph, Germany). Then, 10 ml of ethanol and 4 ml of poly(ethylene glycol) (average Mn400) were added to these mixtures and re-homogenized for another 5 min. A control coating mix (without sonorensin/nisin) was prepared in a similar manner. The LDPE films (15 cm * 10 cm) were fixed on glass plates and 15 ml of the sonorensin and nisin mixtures, separately prepared, were then applied on the prepared film to have sonorensin/nisin at 20 μg/cm^2^. The control mixture was also casted on LDPE in a similar manner. Plates were then allowed to dry at 37 ^o^C for 2 h. After 2 h, the LDPE was detached from the glass plate and analyzed for its antimicrobial activity by placing it on BHI soft agar (0.8%) previously spread with *S. aureus* as indicator strain. The treated face of the LDPE film was in contact with agar. The control film was also tested similarly. The plates were then incubated at 30 ^o^C overnight and observed for the presence or absence of zone of inhibition around the films in a lawn of bacterial cells.

### Antimicrobial activity of sonorensin coated films during the storage of meat products

The developed packaging films were used in challenge tests of control of *L. monocytogenes* and *S. aureus* growth during the storage of meat products. Following superficial spiking of chicken meat pieces with 2 ml suspension of *L. monocytogenes* and *S. aureus* at 1.5 × 10^6^ CFU/ml, the pieces were packed with the active films and stored at 4 ^o^C. Meat pieces packed with untreated films were included in the analysis as controls. After regular intervals of storage, the pieces were observed for the appearance of visible growth of bacteria and obnoxious smell.

In another set of experiments the developed packaging films were used for preventing the spoilage during the storage of vegetables. Fresh tomatoes were packed with the active films and stored at 4 ^o^C. Tomatoes packed with untreated films were included in the analysis as controls. After regular intervals of storage, the tomatoes were observed for the appearance of visible signs of spoilage and rottenness.

### Statistical analysis

All experiments were performed in triplicate and repeated in three independent experiments. The results were presented as the mean ± standard deviations. Statistical significance of difference between the control and the test samples was determined using ANOVA- test and *p* value < 0.005 was considered as significant.

## Conclusions

Sonorensin, a bacteriocin from *B. sonorensis* MT93, was found to be effective against non-multiplying cells of *S. aureus* indicating its potential for antimicrobial therapy and it did not show activity against normal mammalian cells. Sonorensin also effected the inhibition of biofilm formation. The mode of action of sonorensin as revealed by flow cytometry and SEM is the damage of bacterial membrane unlike most of the antibiotics that target cellular components and hence more prone to bacterial resistance. Furthermore, sonorensin, predicted to be the first bacteriocin of subfamily of heterocycloanthracin, demonstrated its efficacy as food biopreservative as the packaging films activated with sonorensin showed preservative effect on food products. Thus, sonorensin could prove to be promising antibiofilm agent as well as natural food biopreservative.

## Additional Information

**How to cite this article**: Chopra, L. *et al.* Sonorensin: A new bacteriocin with potential of an anti-biofilm agent and a food biopreservative. *Sci. Rep.* **5**, 13412; doi: 10.1038/srep13412 (2015).

## Supplementary Material

Supplementary Information
