# Supplementary material for: Sonorensin: A new bacteriocin with potential of an anti-biofilm agent and a food biopreservative
Source: Sci Rep. 2015 Aug 21;5:13412. doi: 10.1038/srep13412 (PMC4544038; doi:10.1038/srep13412)
Supplement: Supplementary Information [file srep13412-s1.pdf]

Supplementary Information

Sonorensin: A new bacteriocin with potential of an anti-biofilm agent and a food biopreservative

Lipsy Chopra, Gurdeep Singh, Kautilya Kumar Jena and Debendra K. Sahoo*

Biochemical Engineering Research and Process Development Centre

CSIR-Institute of Microbial Technology, Sector-39A

Chandigarh-160036, INDIA

* Corresponding author: debsahoo@imtech.res.in

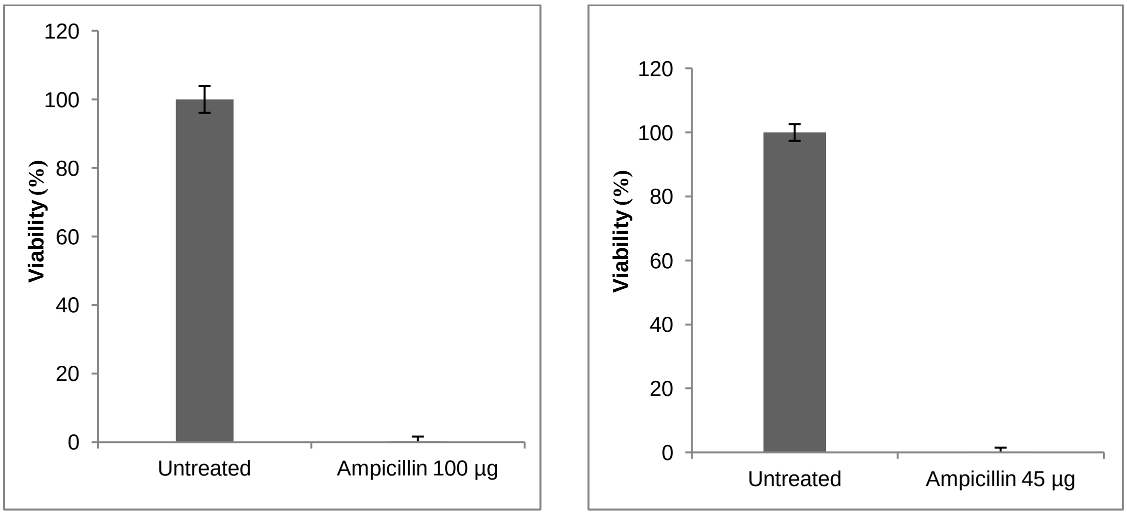

a **b**

Supplementary Figure S1: Cell viability of vegetative cells in the presence of ampicillin (a) *E. coli* and (b) *S. aureus*. The experiments were carried out three times in triplicate. The results were presented as mean ± SD and differences between the control and treated samples were statistically significant (n=3) ($p < 0.005$)

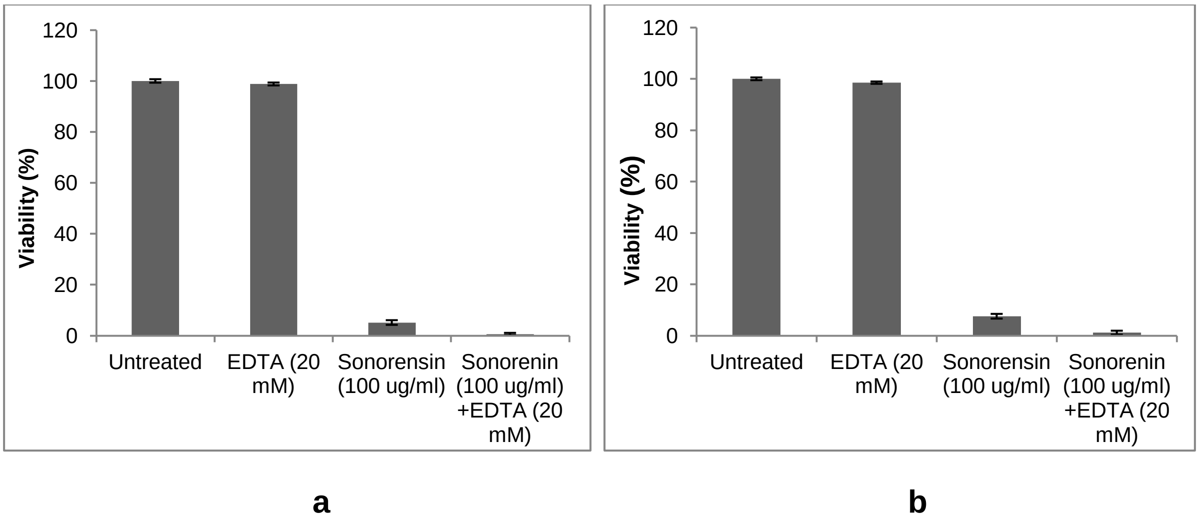

Supplementary Figure S2: Effect of 20 mM EDTA on sonorensin activity against (a) vegetative cells (b) non- multiplying cells of *E.coli*. The inhibitory activity of sonorensin increased in the presence of EDTA. The experiments were carried out three times in triplicate. The results were presented as mean ± SD and differences between the control and treated samples were statistically significant (n=3) ($p < 0.005$)

Supplementary Methods

Effect of EDTA on sonorensin activity

E. coli cells (~2.1 X 10^5) were resuspended in one tube of each of the following: (i) PBS buffer (pH 7.0) alone, (ii) 20 mM EDTA in PBS buffer (pH 7.0), (iii) Sonorensin (100 µg/ml) in PBS buffer (pH 7.0), and (iv) Sonorensin (100 µg/ml) and 20 mM EDTA in PBS buffer (pH 7.0). Cell suspensions were incubated for 2 h at 37 °C and 200 RPM. The treated cells were harvested by centrifugation (5 min), washed twice with PBS buffer, and spread on plates of BHI agar. Plates were incubated at 37 °C for 24 h, and CFU/ml was estimated.

Haemolytic activity assay

Fresh human blood was collected in BD vacutainer and layered on histopaque- 1077. It was then centrifuged for 30 min at 400 × g , the RBCs obtained as pellet were washed three times with 0.9 % (w/v) and finally resuspended in 0.9 % NaCl. Then, 95 µl aliquots of the RBCs suspension were incubated with 5 µl of purified sonorensin at various concentrations for 2 h (50 µg/ml – 500 µg/ml) at 37 °C with gentle mixing. The samples were then centrifuged and the absorbance of the supernatant was measured at 415 nm. Complete lysis was measured by suspending RBC's in 1 % Triton X-100. Percent haemolysis was calculated as:

Percentage haemolysis = $100[(A-A_0)/(A_t-A_0)]$

Where A represents absorbance of peptide sample at 415 nm and A_0 and A_t represent zero percent and 100 % haemolysis determined in PBS and 1 % Triton X-100, respectively.

Cytoplasmic membrane permeability

Bacterial cells, collected in mid-log phase and suspended in M9 minimal medium, were incubated at 37 °C for 8 h with lactose as the sole carbon source. At concentration of 0.3 A_{600} nm (≈1.0×10^6 CFU/ml), 200 µl of bacterial suspension was added into 96-well plate followed by addition of 10 µl ONPG (30 mM) added to each well. Then, 10 µl of sonorensin and nisin were added (50 µg/ml) to each well and the plates were incubated with gentle shaking at 37

°C. The hydrolysis of ONPG to O-nitrophenol over time was monitored at 405 nm with a microplate reader (Biotek, USA).
